# Using a Large Margin Context-Aware Convolutional Neural Network to Automatically Extract Disease-Disease Association from Literature: Comparative Analytic Study

**DOI:** 10.2196/14502

**Published:** 2019-11-26

**Authors:** Po-Ting Lai, Wei-Liang Lu, Ting-Rung Kuo, Chia-Ru Chung, Jen-Chieh Han, Richard Tzong-Han Tsai, Jorng-Tzong Horng

**Affiliations:** 1 Department of Computer Science National Tsing Hua University Hsinchu Province of China Taiwan; 2 Department of Computer Science & Information Engineering, National Central University Taoyuan Province of China Taiwan; 3 Department of Bioinformatics and Medical Engineering, Asia University Taichung Province of China Taiwan

**Keywords:** deep learning, disease-disease association, biological relation extraction, convolutional neural networks, biomedical natural language processing

## Abstract

**Background:**

Research on disease-disease association (DDA), like comorbidity and complication, provides important insights into disease treatment and drug discovery, and a large body of the literature has been published in the field. However, using current search tools, it is not easy for researchers to retrieve information on the latest DDA findings. First, comorbidity and complication keywords pull up large numbers of PubMed studies. Second, disease is not highlighted in search results. Finally, DDA is not identified, as currently no disease-disease association extraction (DDAE) dataset or tools are available.

**Objective:**

As there are no available DDAE datasets or tools, this study aimed to develop (1) a DDAE dataset and (2) a neural network model for extracting DDA from the literature.

**Methods:**

In this study, we formulated DDAE as a supervised machine learning classification problem. To develop the system, we first built a DDAE dataset. We then employed two machine learning models, support vector machine and convolutional neural network, to extract DDA. Furthermore, we evaluated the effect of using the output layer as features of the support vector machine-based model. Finally, we implemented large margin context-aware convolutional neural network architecture to integrate context features and convolutional neural networks through the large margin function.

**Results:**

Our DDAE dataset consisted of 521 PubMed abstracts. Experiment results showed that the support vector machine-based approach achieved an F1 measure of 80.32%, which is higher than the convolutional neural network-based approach (73.32%). Using the output layer of convolutional neural network as a feature for the support vector machine does not further improve the performance of support vector machine. However, our large margin context-aware-convolutional neural network achieved the highest F1 measure of 84.18% and demonstrated that combining the hinge loss function of support vector machine with a convolutional neural network into a single neural network architecture outperforms other approaches.

**Conclusions:**

To facilitate the development of text-mining research for DDAE, we developed the first publicly available DDAE dataset consisting of disease mentions, Medical Subject Heading IDs, and relation annotations. We developed different conventional machine learning models and neural network architectures and evaluated their effects on our DDAE dataset. To further improve DDAE performance, we propose an large margin context-aware-convolutional neural network model for DDAE that outperforms other approaches.

## Introduction

### Background

The origin and treatment of disease is an important research field in the life sciences, covering a wide range of research topics such as comorbidity, complication, genetic disorder, drug treatment, and adverse drug reaction. As disease is involved in many areas, new scientific findings are frequently made or updated.

**Figure 1:**
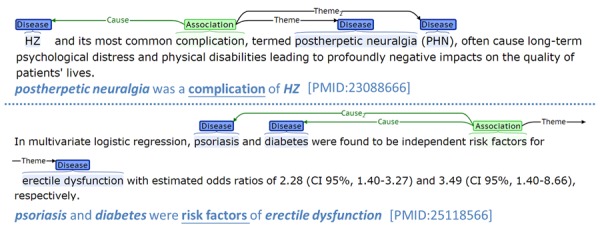
Disease-disease association extraction examples.

Disease-disease association (DDA) is an important research topic in the biomedical domain [1-5]. The influence of one disease on others is wide ranging and can manifest in any patient. Diabetes, for example, may cause macrovascular diseases [6], such as cardiovascular disease [7] and cerebrovascular disease [8]. Treating a disease without consideration of potential DDAs may result in poor treatment outcomes. Therefore, DDAs are often a prime concern for researchers and doctors involved in drug discovery and disease treatment. Figure 1 illustrates examples of DDAs in the literature (refer to Multimedia Appendix 1 for more examples, including comorbidity, complications, general associations, and risk factors). There have been several studies attempting to generate disease connectivity networks [3-5]. However, the enormous and rapidly growing disease-related literature has not been utilized.

Finding DDA in the literature is a time-consuming and challenging task for researchers. First, there are huge numbers of DDA papers to sort through, and existing search engines, such as PubMed, do not mark up all relevant disease mentions in search results. Although there are text-mining tools available that could automatically identify diseases [9-11], genes [10,12,13], chemicals [14,15], and associations among them [16-22], they have not been integrated into a single interface to assist researchers in searching through the latest DDA findings. The main obstacle in creating a DDA extraction (DDAE) system is the lack of a relevant dataset. Moreover, only a few text-mining approaches [23] are suitable for extracting DDA.

In this study, we compiled a DDAE dataset consisting of 521 annotated PubMed abstracts. As it is hard for a human annotator to distinguish one DDA type from another without reading a broader context, such as a whole paragraph, we therefore annotated only 3 DDA types: positive, negative, and null associations:

1. Positive associations include comorbidity, complications, physical associations, and risk factors.
2. Negative associations are counted when the text clearly states that there is no association between 2 diseases.
3. Null associations are annotated when 2 diseases co-occur in a sentence, but no association is stated, suggested, or apparent.

In this study, we formulated DDAE as a supervised machine learning (ML) classification task in which, given a sentence containing a disease pair, the goal was to classify the pair into one of the DDA types. For classification, we employed 2 machine learning models, support vector machine (SVM) [24] and convolutional neural network (CNN) [25]. We compared different combinations of SVM and CNN to maximize performance, arriving at a novel neural network architecture, which we termed as large margin context-aware CNN (LC-CNN). LC-CNN achieved the highest F1 measure of 84.18% on our DDAE test set.

### Related Work

In this section, we first review published disease annotation datasets. Then, we briefly review different methods of relation extraction in biomedical domains.

#### Disease Annotation Datasets

Before identifying DDAs, we have to identify diseases in the text first. Fortunately, there are many datasets for developing such disease name recognition and normalization systems. The National Center for Biotechnology Information (NCBI) disease dataset [26] is the most widely used. For instance, Leaman and Lu [9] proposed a semi-Markov model trained on an NCBI disease dataset that achieved an F1 measure of 80.7%. However, DDAs are not annotated in the NCBI dataset abstracts, limiting its usefulness for the DDAE task.

As DDAs can give insights into disease etiology and treatment, many studies focus on generating DDA networks [1-5]. For example, Sun et al [4] used disease-gene associations in the Online Mendelian Inheritance in Man [27] to predict DDAs with similar phenotypes. Bang et al [3] used disease-gene relations to define disease-disease network, and the causalities of disease pairs are confirmed through using clinical results and metabolic pathways. However, the constructed networks lack text evidence and therefore cannot be used to develop a DDAE dataset.

Xu et al [23] proposed a semisupervised iterative pattern-learning approach to learn DDA patterns from PubMed abstracts. They constructed a disease-disease risk relationship knowledge base (dRiskKB) consisting of 34,000 unique disease pairs. However, there are some limitations of dRiskKB that make it hard to use in developing DDAE systems. First, dRiskKB only provides positive DDA sentences. Owing to the lack of negative instances, it cannot be used to train ML-based classifiers. In addition, as the development of dRiskKB is based on a pattern-learning approach, it only includes DDA sentences with very simple structures and thus is not ideal for training a DDA system capable of analyzing complicated sentences.

To solve the above problems, we developed a DDAE dataset. Our dataset was different from dRiskKB in 3 aspects. First, our DDAE dataset contained positive, negative, and null DDAs. Second, it did not use patterns to annotate DDAs and therefore included DDA sentences with more complex expressions. Finally, it annotated DDAs in the entire abstract, allowing an ML-based classifier to use document-level features.

#### Relation Extraction

Rule-based approaches are commonly used in new domains or tasks that do not have large-scale annotated datasets. Lee et al’s [28] approach is an example. They extracted protein-protein interactions (PPIs) from plain text using handcrafted dependency rules. Their approach did not require a training set, but it achieved a high precision of 97.4% on the Artificial Intelligence in Medicine (AIMed) dataset [29]. However, it was difficult for them to create rules that can extract all PPIs, and their system, therefore, achieved a low recall of 23.6%. Moreover, Nguyen et al [30] used predicate-argument structure (PAS) [31] rules to extract more general relations including PPI and drug-drug interaction. Their rules detected PPIs by examining where relation verbs and proteins are located in the spans of predicates and arguments. Their approach required less effort to design rules and was able to adapt to different relation types. Compared with Lee et al’s system, it achieved a higher recall of 52.6% on the AIMed dataset but a lower precision of 30.4%.

ML-based approaches can usually achieve relatively higher performance than rule-based ones. For instance, Zhang et al [32] used hybrid feature–based and tree-based kernels implemented with SVM-LIGHT-TK [33] for PPI extraction. The feature-based kernel uses SENNA (Semantic/syntactic Extraction using a Neural Network Architecture)’s pretrained word-embedding model [34]. In the tree-based kernel configuration, the sentence dependency structure is used as input. The structure is decomposed into substructures and then transformed into one-hot encoding features for SVMs. Zhang et al’s approach achieved an F score of 69.7% on the AIMed dataset, which is higher than Lee et al’s 26.3% and Nguyen et al’s 38.5%.

In addition to sentence-level features, document-level features are also useful in relation extraction. Peng et al [17] proposed an SVM-based approach for document-level chemical-disease relation (CDR) extraction. They used statistical features, such as whether a chemical or disease name appears in the title, to classify document-level chemical-disease pairs. By adding the features, they improved their F score from a baseline of 46.82% to 57.51% on the BioCreative V CDR dataset [35]. Our LC-CNN is partly inspired by Peng et al’s [17] statistical features; our context vector adopts document-level features for sentence-level DDA classification.

Although the abovementioned feature-based approaches have made gains in many relation extraction tasks [36-38], it is difficult to find novel features to further improve performance. Several researchers are exploring deep learning approaches as a way forward. For instance, Peng and Lu [39] proposed a multichannel dependency-based CNN model (McDepCNN). McDepCNN uses 2 channels to represent an input sentence. One is the word-embedding layer, whereas the other is the head-word-embedding layer. Each embedding layer concatenates pretrained word-embedding vectors, one-hot encodings of part of speech, chunks, named entity labels, and dependency words. In PPI prediction, Peng and Lu’s CNN model achieved F scores of 63.5% on AIMed and 65.3% on BioInfer.

For drug-drug interaction extraction, Lin et al [20] proposed a syntax CNN (SCNN) that integrates syntactic features, including words, predicates, and shortest dependency paths into a CNN. They trained their model with word2vec [40] and the Enju parser [31]. The Enju parser breaks the sentence into PASs, and non-PAS words or phrases are removed. The pruned sentences are then used to train the word-embedding model. Their approach achieved an F score of 68.6% on the 2013 DDIExtraction dataset.

Our LC-CNN was also inspired by Zhao et al’s [20] SCNN architecture with 3 main differences. First, we replaced the log loss function with the hinge loss function. Second, SCNN uses a fully connected layer for traditional features before merging them with the CNN’s output. However, LC-CNN directly merges the CNN’s output with traditional features. Finally, SCNN’s traditional features only use sentence-level information, whereas LC-CNN also uses both sentence-level and document-level features.

## Methods

### Study Process

In this section, we have first described the process of DDAE dataset construction. We then introduced our LC-CNN architecture in subsection *The Neural Network Architecture*. Further, we described each layer of LC-CNN in subsection *Composite Embedding Vector* to *Output Layer of Combined Sentence and Context Vector*. Finally, we introduced backward propagation for learning parameters of each layer.

### Dataset Construction

**Figure 2:**
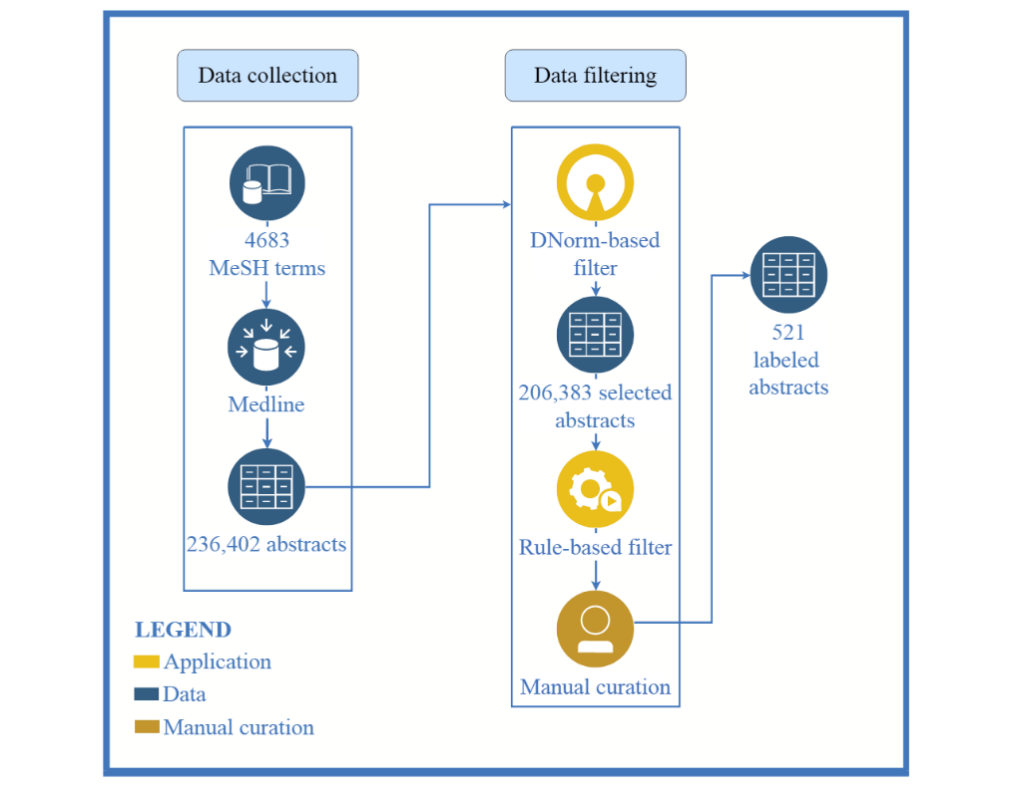
Disease-disease association extraction dataset construction process. MeSH= Mesdical Subject Headings.

The process of DDAE dataset construction is illustrated in Figure 2. Our DDAE dataset consisted of abstracts found in PubMed. To generate PubMed search queries related to DDA, we selected all disease nodes of the MeSH [41] tree whose tree number prefix starts with *C* and *F*, indicating diseases. We then selected any nodes related to human diseases. This produced a list of approximately 4700 disease names, which we then used to retrieve 236,000 abstracts whose titles or content contain one or more query terms.

As some of these abstracts do not contain any DDAs, we used simple heuristic rules and a disease name recognizer/normalizer to select abstracts with a higher likelihood of containing DDAs. The process was as follows:

1. We selected only abstracts published from 2013 to 2017.
2. We used DNorm [42] to annotate disease mentions and their Medical Subject Heading (MeSH) IDs in these abstracts.
3. To ensure that the selected abstracts contain rich DDAs for training classifier, we removed abstracts that have fewer than 3 sentences that contain at least two different disease MeSH IDs.
4. To ensure the selected abstracts contain at least one DDA, we applied a DDA-adapted version of Lee et al’s [28] dependency tree-based relation rules and removed any abstract not matched by any rule.
  5. We randomly selected 521 abstracts from the remaining abstracts for annotation.

For the manual annotation step, we employed 2 biomedical specialists. Annotator 1 is a PhD candidate in a bioinformatics program, whereas Annotator 2 is a full-time research assistant in a hospital. Both have at least 6 years of biomedical experience. After agreeing on initial annotation guidelines (refer to Multimedia Appendix 1—Annotation Guideline), they used the brat rapid annotation tool [43] to annotate 10 abstracts and then compare results. In the first independent annotation processing, Cohen kappa value was 34%. Once both annotators agreed that all annotations that indicate consistency is satisfactory, they each annotated all remaining abstracts. Thus, each abstract was annotated independently twice. Inconsistent annotations were resolved afterward through discussion. The final Cohen kappa value was 76%.

### The Neural Network Architecture

**Figure 3:**
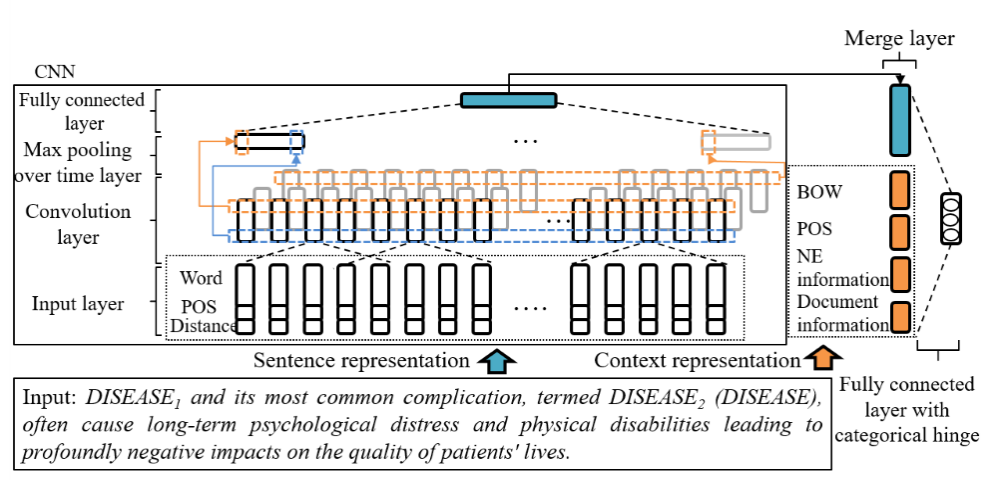
Large margin context-aware convolutional neural network (LC-CNN) architecture. BOW: Bag of words; POS: Part of speech; NE: Named Entity.

We formulated relation extraction as a classification problem in which, given a sentence containing a mention pair, the goal was to classify the pair into one of relation types. For classification, we propose an LC-CNN architecture as illustrated in Figure 3. The network is fed input in 2 forms: sentence representation and context representation (CR). Sentence representation is a *n_emb_* x *T* matrix representing the sentence. *n_emb_* and *T* are the length of composite embedding vector and the length of the sentence, respectively. The sentence representation uses only word embedding, part of speech (POS) encoding and Named Entity (NE) distance information, and parameters are learned through the next CNN and max-pool layers, which outputs an *m*-dimension sentence-level feature vector. The CR is a feature-rich *n*-dimension vector containing both syntactic and document-level features, such as whether the disease pair also appears in the title. Next, the *m*-dimension vector and the *n*-dimension vector are concatenated to form the final feature vector with (*m*+*n*) dimension. To compute the confidence of each relation type, the feature vector is fed into a fully connected layer, where we use a linear activation function with categorical hinge loss [44]. The output layer is a three-dimensional vector, with each dimension value representing the confidence of a predefined relation type.

### Composite Embedding Vector

In a sentence, each word is represented as a composite embedding vector, as shown in Figure 3 (or in Multimedia Appendix 2). A composite embedding vector consists of 3 parts: word embedding, POS one-hot coding, and the distance between the word and disease pair. A matrix represents a sentence. The matrix contains the composite embedding vectors in the sentence, each placed in the order in its row. The sentence matrix is a matrix of size *n_emb_* x *T*, where*n_emb_* is the dimension of the composite embedding vector and *T* represents the maximum length of the sentence in the dataset.

#### Word Embedding

The embedding of a word is a mapping of the word to a vector of real values. Generally, the word embeddings of semantically similar words are closer together in the vector space. Word embedding learned by neural networks has been demonstrated to be able to capture linguistic regularities and patterns in language models [40]. Therefore, it is commonly used in features in popular NN approaches, such as CNN [20,39] and long-short term memory (LSTM) [19]. In general, word embeddings are learned from large corpora such as Wikipedia or PubMed. For example, Pyysalo et al [45] applied word2vec to learn word embeddings from different texts, including Wikipedia, PubMed abstracts, and PubMed Central full-text papers, and developed a word-embedding lookup dictionary. Here, we employed their dictionary to generate word embeddings.

#### Part of Speech

The embedding of a word is a single vector and, therefore, cannot fully represent the multiple syntactic/semantic roles of a word like *good*, which can be either an adjective or a noun. The POS feature is designed to provide syntactic information (part of speech) to help the model separate the different semantic senses of a word. We used Zhao et al’s [20] approach, in which similar POSs are assigned to the same group. We divided POSs into 11 groups, including adjectives, adverbs, articles, conjunctions, foreign words, interjections, nouns, prepositions, pronouns, punctuation, and verbs. If a word belongs to a POS group, the corresponding bit value will be 1; otherwise, it will be 0.

#### Named Entity Distance

Zeng et al [46] proposed the use of NE distance (position features) to improve a CNN by keeping track of how close words are to the target nouns. We adopted their NE distance in this study. The NE distance feature is a two-dimensional vector (*d_1_*, *d_2_*). *d_1_* and *d_2_* represent the distance (number of words) between the current word and the first and second diseases of the pair.

### Context Representation Layer

Contextual information, such as pair and document information, is very useful for classification and has been widely used in previous research. The purpose of using contextual representation is to introduce traditional contextual features into a neural network architecture through simple representation. We can then apply the fully connected layer to the context vector to obtain a condensed vector that combines 2 different representations.

Here are the features used in our contextual representation (refer to Multimedia Appendix 3 for more details).

#### Bag of Words

Word embedding has been shown to represent abstract information about words. However, word embedding can sometimes change the original meaning of a word. For example, *not* usually appears in negative relation statements. However, in the word2vec model trained on news, the 3 words most similar to *not* are *do*, *did,* and *anymore*. This violates our intuition that *don’t*, *doesn’t,* and *isn’t* are more similar to *not* in the relation statement. As the embedded vector words of certain words may differ in the news and biomedicine domains, we use BOW features for context vector. Our BOW features include unigram, bigram, and surrounding diseases.

#### Part of Speech

The POS tags are commonly used for relation extraction. We used one-hot encoding to represent each word’s POS tag type.

#### Named Entity Information

The number of diseases is useful when classifying relations. We used 3 different features to capture information, including the following:

1. The number of tokens between disease pairs.
2. The number of diseases between disease pairs.
3. The number of diseases in the sentence.

#### Document-Level Information

Biological papers usually follow a certain flow to describe their experimental and scientific findings. Therefore, article structure often provides valuable information about relations. We used 2 types of document-level feature, core pair and pair location. The core pair features indicate whether the current disease is a top-3 frequent disease pair in the article. The 3 most frequent pairs are treated as 3 features. The pair location feature is used to indicate the position of the sentence containing the relation in the article. If the sentence is the article title, it usually contains the subject of the article, which might be a relation investigated in the paper. Similarly, if the sentence is the last sentence of the abstract, it may summarize the main scientific discovery of the article. We used 3 binary features to represent relation pairs that appear in the title, the first sentence of the abstract, the last sentence of the abstract.

### Output Layer of Combined Sentence and Context Vector

We used *m_concat_* = [sr cr] to represent the concatenation of sentence representation sr and context representation cr. The size of the vector *m_concat_* is *n_concat_* = *n_sr_* + *n_cr_*. We then applied a fully connected layer to *m_concat_* to obtain a 3D vector *out*, each value of which refers to the confidence of a predefined category.

*out* = *W_out_* x *m_concat_* + *Bias_out_*

*W_out_* is a matrix with a size of *n_out_* x *n_concat_* and *Bias_out_* is a bias matrix with a size of *n_out_* x 1. *n_out_* is the number of predefined categories. out is the output of this fully connected layer and is defined as matrix *W_out_* multiplied by matrix *m_concat_*, plus bias *Bias_out_* Therefore, the size of out is *n_out_* x 1. *out* is the final output of the prediction, and each dimension value of out refers to the score of its predefined category. out is calculated by a linear activation function, the values of out could be R × R × R.

### Backward Propagation With Large Margin Loss

We used the following parameters:

1. k weight matrices, convWf each with a size of ne x f. Here, ne is the size of the input embedding vector of a word, and f is the window size of the filter.
2. k biases, convBf, each with size of ne x 1.
3. Weight matrix Wsr with a size of nsr x npool. Here, nsr is the output dimension of sentence vector and a hyperparameter.
4. Bias Biassr with a size of nsr x 1.
5. Weight matrix wout with a size of nout x nconcat. Here, nout is the number of relation types.
6. Bias BiasmaxF with a size of nout x 1.

In forward propagation, given those parameters, we calculated out with the methods mentioned in section *The Neural Network Architecture* to *Context Representation Layer*. In backward propagation, gradient descent is used to learn these parameters through minimizing the hinge loss of out. Given a sentence and its disease-disease pair, we defined a vector y as the pair’s relation label vector. *y* is a 3D vector, and each dimension value of y represents the score of one relation type. According to the definition of hinge loss [44], the value is either -1 or 1. *1* means that the pair belongs to the relation type, whereas *–1* means it does not. Therefore, one value of the 3D vector must be *1*, and the others must be *–1*. For instance, the 3 vectors <1, –1, –1>, <–1, 1, –1>, and <–1, –1, 1> indicate that 3 vectors are *Positive*, *Negative*, and *Null*, respectively. We used the hinge loss function to evaluate the loss between prediction out and its truth label *y*; a larger loss indicates a larger gap between *out* and *y*. The hinge loss function is defined as follows:

loss(*out, y*)=sum*_i_*_=1 to_*_nout_*(max(1 - *y_i_* * *out_i_*, 0))/*n_out_*

Here, *y_i_* is the *i*-th dimension value of *y*. *out* is calculated by using forward propagation (sections *The Neural Network Architecture* to *Context Representation Layer*), and each dimension value of *o* refers to the prediction score of one predefined relation type. *out_i_* is the *i*-th dimension value of out. *out_i_* belongs to *R*. If *out_i_* is a positive value, then the pair may be the *i*-th relation type. Otherwise, if *out_i_* is a negative value, then the pair is less likely to be the *i*-th relation type.

In the equation, 1 is the value of the decision boundary. Ideally, *y_i_* * *out_i_* will be larger than the decision boundary value. If *y_i_* and *out_i_* have the same sign, then *y_i_* * *out_i_* will be a positive value belong to R. If *y_i_* * *out_i_* is larger than the decision boundary value 1, then the loss(*out, y*) must be 0. If *y_i_* * *out_i_* is smaller than the decision boundary value 1, then the loss(*out, y*) must be 1 - *y_i_* * *out_i_* which is equal to the cost. If *y_i_* and out_i_ are different signs, then *y_i_* * *out_i_* will be a negative value ε R. Therefore, the loss(*out, y*) is a value greater than 1.

Given the training set

T={(*x^(i)^*,*y^(i)^*) | *i* = 1,…, *N* },

*x^(i)^* is the *i*-th instance in the training set, *y^(i)^* is its label vector, and *N* is the number of training instances. Weight learning consists of the following optimization:

argmin*_convWf, convBf, Wst, Biassr, Wout, Biasout_* loss(*out*,*y*)

Finally, mini-batch stochastic gradient descent [47] is applied to update the learned parameters in each iteration.

## Results

### Dataset

Currently, there are no available annotated datasets for training DDA extraction systems. To create one, we used our DDAE dataset development process, described in section *Dataset Construction*. The DDAE dataset consists of 521 annotated abstracts. After annotation, we used Cohen kappa coefficient to evaluate annotation consistency. The final kappa value is 76%, suggesting a high level of agreement.

For the experiments in this study, we divided our DDAE dataset into a training set of 400 abstracts and a test set of 121 abstracts. Before testing, we tuned the hyperparameters on one-third of abstracts randomly chosen from the training set called tuning set. Finally, our classifiers were trained on the whole training set and evaluated on the test set. A summary of the final DDAE dataset is shown in Table 1.

**Table 1:** Summary of disease-disease association extraction dataset.

Type	Training set, n	Test set, n	Total, n
Abstracts	400	121	521
Sentences	4820	1549	6369
Diseases	9522	2824	12,346
Total pairs	9086	2419	11,505
Positive pairs	2538	623	3161
Negative pairs	126	35	161
Null pairs	6422	1761	8183

### Experiment Setup

We conducted 3 experiments to evaluate our LC-CNN. The first experiment was designed to measure the effects of different NN architectures and ML models. In the second experiment, we evaluated the effects of different approaches combining context features with NN methods. In the third experiment, we evaluated the effects of different word embeddings. The hyperparameters are listed in Multimedia Appendix 4. The performances of experiments on the tuning set can be found in Multimedia Appendix 5.

Our system is implemented on TensorFlow with Keras and runs on an Nvidia GTX 1080ti GPU. The process used in our experiments to generate the word-embedding model can be found in Multimedia Appendix 6.

### Evaluation Metric

**Figure 4:**
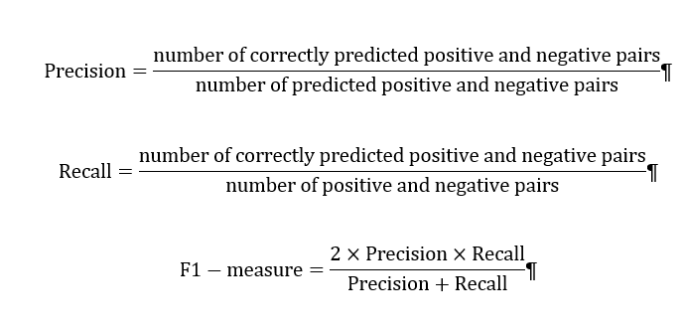
Precision and recall formula.

We used the F1 measure to evaluate system performance. The precision and recall are defined as given in Figure 4.

### Experiment 1—Performance Comparison With Other Models

The performance comparison between LC-CNN and different methods is listed in Table 2. It shows the performances on the tuning and test sets. The NN models (models 1 to 3) use only sentence representation. The CR_cross-entropy_ and SVM methods use only CR. CR_cross-entropy_ is implemented using a single hidden fully connected layer with the context vector as its input layer, and its architecture can be found in Multimedia Appendix 7. Furthermore, we also compared LC-CNN with LSTM and bidirectional LSTM (BiLSTM) models. They have been used in many relation extraction tasks, such as those seen in the studies by Hsieh et al and Zhao et al [19,48]. In our experiment, we were surprised to find that LSTM achieved the lowest F1 measure (65.02%) on the test set among all tested models. Furthermore, we also evaluated the performance of SCNN, Bidirectional Transformers for Language Understanding (BERT) [49], and BioBERT [50]. As we would like to compare the architecture of SCNN with LC-CNN, LC-CNN and SCNN use the same sentence representation, CR, and hinge loss function. The architecture of SCNN is illustrated in Multimedia Appendix 8.

As shown in Table 2, NN models trained on the entire training set (models 1 to 3) performed worse on the test set than on the tuning set. One potential reason is that the selected hyperparameters and parameters may be less likely to find unseen data, which could cause the hyperparameters and parameters of the NN models to overfit the tuning set. This problem is especially obvious in the LSTM and BiLSTM models. In contrast, CR_cross-entropy_, SVM, and LC-CNN models trained on the entire training set with context information performed better on the test set than on the tuning set.

Furthermore, as shown in Table 2, CNN and CR_cross-entropy_ performed similarly on the tuning set. The F1 measures of CNN and CR_cross-entropy_ were 75.35% and 75.76%, respectively. CNN’s recall rate was better than CR_cross-entropy_’s recall rate by 2.84%, whereas CR_cross-entropy_’s precision was 3.95% higher than that of CNN. This may be because the document feature provides CR_cross-entropy_ with the information on the entire document, thus causing the model to generate fewer false positive cases. As CNN does not directly encode document information, it predicts more FPs. However, as CNN does not use any particular feature to separate positive, negative, and null relation pairs, it may be able to extract potential positive and negative pairs missed by CR_cross-entropy_, resulting in higher recall rates. In addition, the SVM and CR_cross-entropy_ use the same input features, but SVM mainly uses large margin for learning. The result shows that the SVM implemented with LibSVM [24] outperforms the CR_cross-entropy_ by an F1 measure of 2.83%. Moreover, LC-CNN is able to combine the advantages of CNN and SVM to achieve the highest precision/recall/F1 measure among the tested models and outperforms SCNN, BERT, and BioBERT by F1 measures of 3.25%, 2.06%, and 1.91, respectively.

**Table 2:** Performances of different models. P: Precision; R: Recall; F: F1-Measure.

Input	Model	Tuning set			Test set		
		P (%)	R (%)	F (%)	P (%)	R (%)	F (%)
SR^a^	LSTM^b^	65.53	70.15	67.76	66.13	63.95	65.02
SR	BiLSTM^c^	73.78	70.12	71.90	65.16	65.64	65.40
SR	CNN^d^	75.31	75.39	75.35	74.86	71.84	73.32
CR^e^	CR_cross-entropy_	79.26	72.55	75.76	77.78	77.19	77.49
CR	SVM^f^	74.86	81.03	77.86	78.44	82.29	80.32
SR+CR	SCNN^g^	79.23	88.30	83.52	75.31	87.44	80.93
SR+CR	LC-CNN^h^	82.58	87.72	85.07	82.36	85.00	84.18
Sentence+pair	BERT	77.23	80.27	78.72	79.24	85.23	82.12
Sentence+pair	BioBERT	80.22	83.75	81.95	80.24	85.35	82.27

^a^SR: sentence representation.

^b^LSTM: long-short term memory.

^c^BiLSTM: bidirectional long-short term memory.

^d^CNN: convolutional neural network.

^e^CR: context representation.

^f^SVM: support vector machine.

^g^SCNN: syntax convolutional neural network.

^h^LC-CNN: Large margin context-aware convolutional neural network.

### Experiment 2—Effect of Different Uses of Context Information

To demonstrate the advantage of integrating CNN and context information in a single LC-CNN architecture, we evaluated different ways of combining them. The performances of these combinations are shown in Table 3. There are 3 baseline models that use only either CNN or context information. Baselines 1 to 3 are CR_cross-entropy_, SVM, and CNN and are used in Experiment 1. Only CR_cross-entropy_ and SVM use contextual information.

SVM + CNN is an intuitive method in which the output vector of CNN is considered an additional feature vector of SVM, and its architecture is illustrated in Multimedia Appendix 9. As shown in Table 3, the F1-measure of SVM + CNN is significantly lower than that of SVM by 6.98%. One possible reason is that the CNN used in SVM + CNN is adjusted on the tuning set, so it causes the model to overfit CNN predictions, making it difficult to learn feature weights well.

We designed the LC-CNN to learn the model in a single stage. LC-CNN achieves an F1 measure of 84.18% on the test set, which is the highest score among all methods and outperform SCNN. The results showed that LC-CNN can learn CNN and context information well in a single stage.

**Table 3:** Performance of combined classifiers. P: Precision; R: Recall; F: F1-Measure.

Method	P (%)	R (%)	F (%)
Baseline 1 (CR^a^_cross-entropy_)	77.78	77.19	77.49
Baseline 2 (SVM^b^)	78.44	82.29	80.32
Baseline 3 (CNN^c^)	74.86	71.84	73.32
SCNN^d^	75.31	87.44	80.93
LC-CNN^e^	82.36	85.00	84.18
SVM+CNN (2-stage)	74.45	72.26	73.34

^a^CR: context representation.

^b^SVM: support vector machine.

^c^CNN: convolutional neural network.

^d^SCNN: syntax convolutional neural network.

^e^LC-CNN: large margin context-aware convolutional neural network.

### Experiment 3—Effect of Composite Embedding Vectors on Large Margin Context-Aware Convolutional Neural Networks

In our third experiment, we evaluated the effect of different composite embedding vectors on LC-CNN (the effect of different features on LC-CNN can be found in Multimedia Appendix 10). The performance on the test set is shown in Table 4. We compared 3 different word embeddings. The word embeddings of LC-CNN_PubMed_ are from Pyysalo et al [45], who learned them from Wikipedia, PubMed abstracts, and PubMed Central full texts. The word embeddings of LC-CNN_News_ are learned from Google News using word2vec. In contrast, LC-CNN_no pretrain_ does not use any pretrained word embeddings. Its word embeddings are treated as parameters and are learned through training LC-CNN_no pretrain_ on the training set. Moreover, we also evaluated the effect of 3 different embedding features (word embedding, POS, and NE distance) by removing them individually from the LC-CNN_PubMed_.

As shown in Table 4, the model with PubMed word embeddings (LC-CNN_PubMed_) outperformed LC-CNN_News_ and LC-CNN_no pretrain_. In addition, our removal tests indicated that both POS and NE distance have strong impact on performance.

**Table 4:** The effect of different composite embedding vectors on large margin context-aware convolutional neural network performance. P: Precision; R: Recall; F: F1-Measure.

Method	P (%)	R (%)	F (%)
LC-CNN^a^_PubMed_	82.36	85.00	84.18
LC-CNN_news_	79.80	87.36	83.41
LC-CNN_no pretrain_	77.83	86.58	81.97
LC-CNN_PubMed_—POS^b^	80.23	84.26	82.19
LC-CNN_PubMed_—distance	77.68	87.08	82.11

^a^LC-CNN: large margin context-aware convolutional neural network.

^b^POS: part of speech.

## Discussion

### Large Margin Context-Aware Convolutional Neural Network Error Cases Distribution

We randomly sampled approximately 60 error cases of the LC-CNN’s predictions, and their distribution is illustrated in Table 5. FP and FN denote the false positive and false negative cases, respectively. As shown in Table 5, the *symptom/subclass* is a common error category in the FPs, and it contains a ratio of 28% in the sampled error cases. The *symptom/subclass* indicates that a disease is either a subclass or a symptom of another disease in the FP/FN disease pair. For example, an FP case: “Other large-artery aneurysms, including carotid, subclavian, and *iliac artery aneurysms*_DISEASE1_, have also been associated with *Marfan syndrome*_DISEASE2_. --- PMID:23891252” [51].

Here, the *carotid*, *subclavian*, and *iliac artery aneurysms* are 3 *Traumatic syndrome* for *Marfan syndrome*. They are the symptoms of *Marfan syndrome*. The symptom is not included in our DDA definition. Therefore, *iliac artery aneurysms*_DISEASE1_ does not have a relation with the *Marfan syndrome*_DISEASE2._ However, in this case, the keyword phrase *been associated with* makes LC-CNN predict it as positive relation, and thus results in an FP case.

In contrast with the FP cases, the FN cases are relatively sparse, and most of them cannot be categorized. For example, “CONCLUSION: *Cataract*_DISEASE1_, uncorrected refractive error, and fundus diseases are ranked in the top 3 causes of moderate to severe *visual impairment *_DISEASE2_ and blindness in adults aged 50 years or more in rural Shandong Province. --- PMID: 23714032” [52].

In the sentence, *Cataract* is one cause of *visual impairment*; however, the description also lists the other 2 diseases that cause *visual impairment*. For example, “it can be associated with any type of *vision loss*_DISEASE1_ including that related to *macular* *degeneration*_DISEASE2_, *corneal disease*_DISEASE3_, *diabetic retinopathy*_DISEASE4_, and *occipital infarct*_DISEASE5_. --- PMID:24339694” [53].

Here, the LC-CNN correctly identifies the relation between DISEASE1 and DISEASE2. However, it failed to identify the relations between DISEASE1 and the other diseases (DISEASE3, DISEASE4, and DISEASE5).

**Table 5:** The distribution of sampled large margin context-aware convolutional neural network error cases.

Type, category		Description	Ratio (%)
**FP^a^**			
	Symptom/subclass	A disease is a symptom/subclass of another disease	28
	Co-occur	2 diseases co-occur in the sentence	24
	Negation	2 diseases are negative relation	8
	Others	The error cannot be categorized	40
**FN^b^**			
	Simple FN	There is an obvious relation keyword for disease pair	23
	Negation	2 diseases are negative relation	16
	Others	No obvious relation keyword, or the statements of DDA^c^ are too complicated	61

^a^FP: False positive.

^b^FN: False negative.

^c^DDA: disease-disease association.

### The Result of Using Automatic Annotated Disease Mentions

In our experiment, we used the manually annotated disease mentions, which may not reflect the actual performance of the fully automated DDAE task. Hence, we conducted an experiment, in which we used the TaggerOne [9], a state-of-the-art disease mention recognizer/normalizer, to annotate the disease mentions of the test set. Then we used the LC-CNN to extract DDAs from the TaggerOne-annotated test set. As the boundaries of some predicted mentions may be inconsistent with the gold mentions, we used an approximate matching to allow this. In the fully automatic process, the LC-CNN achieved a Precision/Recall/F1 measure of 75.28/55.03/63.57, respectively. The recall is significantly lower because it failed to recognize some diseases. However, the performance is reasonable but 7.08% lower than that of the semiautomatic process (using gold disease mentions).

### Principal Findings

Our objective was to develop a DDAE dataset and a neural network–based approach to extract DDAs. In our experiments, the LC-CNN trained on our dataset achieved an F1 measure of 84.18%. We also compared LC-CNN with common NN models including CNN, Bi-LSTM, and SVM. The results showed that the LSTM and BiLSTM models achieved relatively lower F1 measures of 65.02% and 65.40%, respectively. This may be because the hyperparameters and parameters tend to overfit the training set. The CNN and SVM models achieved relatively higher F1 measures of 73.32% and 77.49%, respectively, but LC-CNN still outperformed all tested methods. In addition, the results showed that the 2-stage *SVM + CNN* model scored significantly lower in terms of F1 than SVM and LC-CNN by 6.98% and 10.84%, respectively. This suggests that simple methods may achieve better results than complex ones. Furthermore, in our experiments, the model with PubMed word embeddings (LC-CNN_PubMed_) outperformed the LC-CNN_News_ and LC-CNN_no pretrain_ models, indicating that PubMed word embeddings may be more compatible with our DDAE dataset.

### Conclusions

In this paper, we proposed a text-mining approach for automatically extracting DDAs from abstracts. We collected disease-related abstracts from PubMed and annotated the first publicly available DDAE dataset consisting of 521 abstracts and 3322 disease-disease pairs. Moreover, to extract DDAs, we used several different ML models, including BiLSTM, CNN, and SVM. We also evaluated the effect of combining CNN and context features. Finally, we implemented a novel neural network called LC-CNN to integrate context features and CNN through the large margin function. Our experiment results showed that LC-CNN achieved an F1 measure of 84.18%, the highest among the tested models.

## Appendix

Multimedia Appendix 1 Annotation Guideline.

Multimedia Appendix 2 Composite Embedding Vector.

Multimedia Appendix 3 Context Representation Layer.

Multimedia Appendix 4 Hyperparameters.

Multimedia Appendix 5 Performances on Tuning Set.

Multimedia Appendix 6 Generating Word Embedding.

Multimedia Appendix 7 Architecture of CRcross-entropy.

Multimedia Appendix 8 Architecture of SCNN.

Multimedia Appendix 9 Architecture of SVM + CNN.

Multimedia Appendix 10 Effect of Different Features on LC-CNN.

Multimedia Appendix 11 DDAE Dataset.

## Abbreviations

BERT: Bidirectional Transformers for Language Understanding
BiLSTM: bidirectional long-short term memory
BOW: bag of words
CDR: chemical-disease relation
CNN: convolutional neural network
CR: context representation
DDA: disease-disease association
DDAE: disease-disease association extraction
dRiskKB: disease-disease risk relationship knowledge base
LSTM: long-short term memory
McDepCNN: multichannel dependency-based convolutional neural network
MeSH: Medical Subject Headings
ML: machine learning
NCBI: National Center for Biotechnology Information
PAS: predicate-argument structure
POS: part of speech
PPI: protein-protein interaction
SCNN: syntax convolutional neural network
SVM: support vector machine
