# Supplementary material for: Using a Large Margin Context-Aware Convolutional Neural Network to Automatically Extract Disease-Disease Association from Literature: Comparative Analytic Study
Source: JMIR Med Inform. 2019 Nov 26;7(4):e14502. doi: 10.2196/14502 (PMC6913619; doi:10.2196/14502)
Supplement: Multimedia Appendix 1 [file medinform_v7i4e14502_app1.pdf]

Multimedia Appendix 1: Annotation Guideline

1. Annotation of Disease Name

The annotation standard of disease name is the same with the NCBI disease corpus [1]. First, diseases are automatically annotated by using a commonly-used disease recognizer, DNorm tool [2], and then only human disease entities are reserved for annotating disease-disease association (DDA). Finally, our annotators will manually remove incorrect disease entity annotations generated from DNorm.

2. The Scope of Disease-Disease Association (DDA)

In our disease-disease association extraction (DDAE) corpus, the annotated DDAs are categorized into two types: **Positive** and **Negative** associations. Their definitions are as follows:

- **Positive association:** include *comorbidity*, *complication*, *physical association*, and *risk factor*, if there are any of the mentioned terms linking a pair of diseases, the type will be annotated as Positive association.
  - comorbidity: A sentence uses *comorbidity* keyword to link the DDA. Eg.

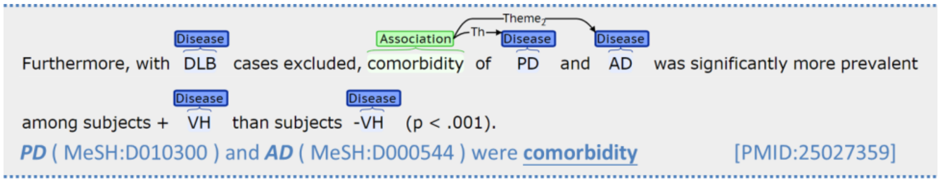

  - complication: *Complication* is mentioned as a bridge of DDA. Eg.

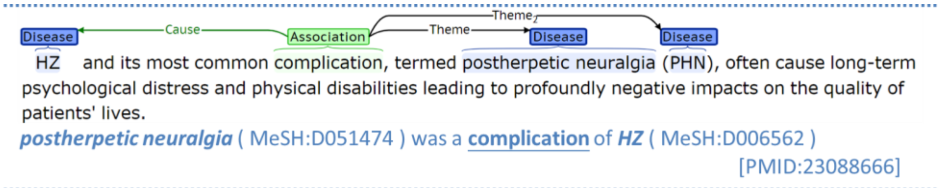

  - physical association: The regular co-occurrence of DDA without additional information is assigned *physical association*. Eg.

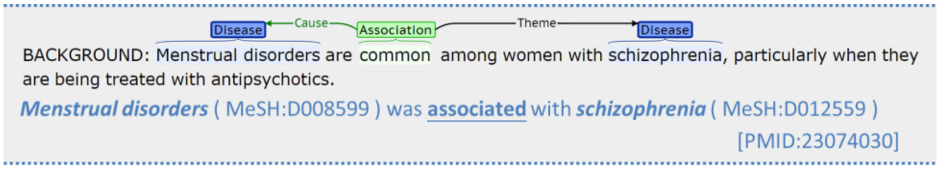

  - risk factor: Risk *factor* is used for DDA when the target disease raises the

possibility of getting another one. For example:

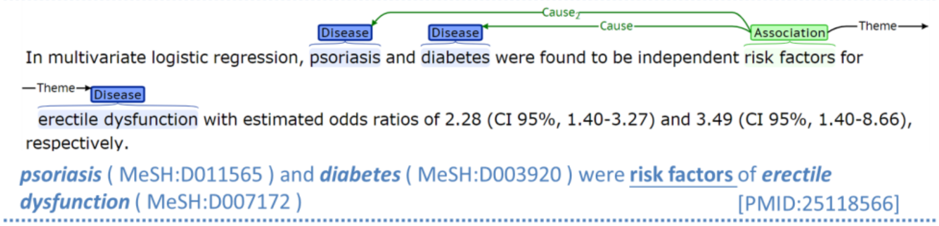

- **Negative association**: Negative association is picked when the text clearly states that there is no association between two diseases. Eg.

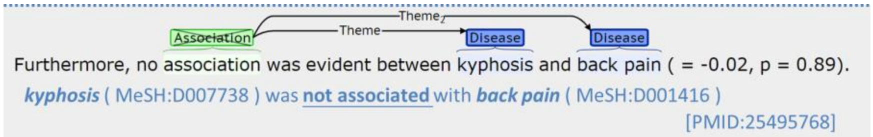

3. Annotation of General DDA:

- **Annotation of Relation Keyword:** annotators have to annotate the evidence keyword(s) of DDA in a given sentence. For example, *"comorbidity"*, *"complication"*, *"symptom"* and *"risk factor"*. If there is no such keyword, the main verb which connects the disease relationship is the next available option. Moreover, once there is no proper verb, the annotators then consider the main adjective or noun which connects the relationship between two diseases.
- **Annotations of Cause and Theme:** If a DDA contains causal relationship, annotators should annotate one disease as Cause and another as Theme. Otherwise, both diseases will **be** labeled as Themes.

4. Annotation of Special DDA Cases:

- **The relationship between Disease$_A$ and Disease$_B$ in/among Disease$_C$ (or … with Disease$_C$):** If a sentence describes the relationship of Disease$_A$ and Disease$_B$ in Disease$_C$, annotators will consider Disease$_C$ as a condition statement and treat it as an additional state. Then only the relationship between Diseases$_A$ and Diseases$_B$ is annotated as DDA. Eg.

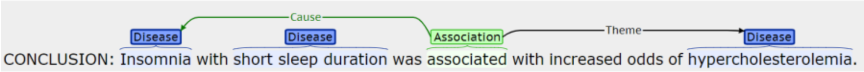

<PMID:26536829>

Above sentence can be simplified as among women who have *"schizophrenia"*, investigating the relationship between *"hyperprolactinemia"* and *"menstrual*

disorders". Therefore annotators will consider *"schizophrenia"* as an additional state and is not related to two diseases.

Similar, the following case:

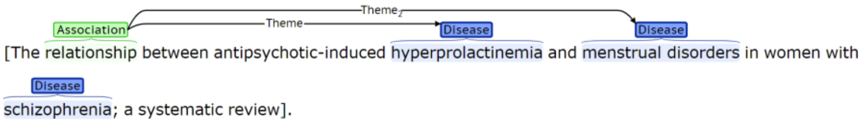

<PMID:23074030>

The *"short sleep duration"* is considered to be an additional state of *"insomnia"*, and *"short sleep duration"* did not directly participate in the association of *"insomnia"* and *"hypercholesterolemia"*.

- **Disease$_A$ is characterized by … Disease$_B$,**

  Disease$_A$ is classified by … Disease$_B$, or

  Disease$_A$ is … Disease$_B$: If a sentence describes the Disease$_A$ is … Disease$_B$, it usually indicates Disease$_A$/Disease$_B$ is a symptom or subcategory of another one Disease$_B$/Disease$_A$. Therefore the association is not taken into consideration. Eg.

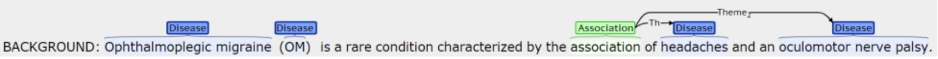

<PMID:23150890>

In above example. *"headaches"* and *"oculomotor nerve palsy"* are symptoms of *"Ophthalmoplegic migraine"*. Therefore, there is no association between *"Ophthalmoplegic migraine"* and two diseases. For more examples:

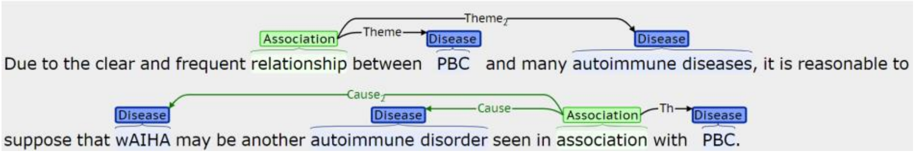

<PMID:26630456>

The phrase *"… **wAIHA** may **be** … **autoimmune disorder** …"*. "*wAIHA*" belongs to a member of *"autoimmune disorder"* so the association between them is neglected.

Reference

[1] R. I. Doğan, R. Leaman, and Z. Lu, "NCBI disease corpus: A resource for disease name recognition and concept normalization," *Journal of Biomedical Informatics*, vol. 47, pp. 1-10, 2014/02/01/ 2014.

[2] R. Leaman, R. I. Dogan, and Z. Lu, "DNorm: disease name normalization with pairwise learning to rank," in *Bioinformatics*, ed, 2013.
