# Supplementary material for: Using a Large Margin Context-Aware Convolutional Neural Network to Automatically Extract Disease-Disease Association from Literature: Comparative Analytic Study
Source: JMIR Med Inform. 2019 Nov 26;7(4):e14502. doi: 10.2196/14502 (PMC6913619; doi:10.2196/14502)

Multimedia Appendix 2 Composite Embedding Vector

In a sentence, each word is represented as a composite embedding vector, as shown in Fig. 1. A composite embedding vector consists of three parts: word embedding, POS one-hot coding, and the distance between the word and the disease pair. A sentence is represented by a matrix that contains the composite embedding vectors in the sentence, each placed in order in its own row. The sentence matrix is a matrix of size T x n_e, where T represents the maximum length of the sentence in the dataset and n_e is the dimension of the composite embedding vector

Fig. 1. Example of composite embedding vector

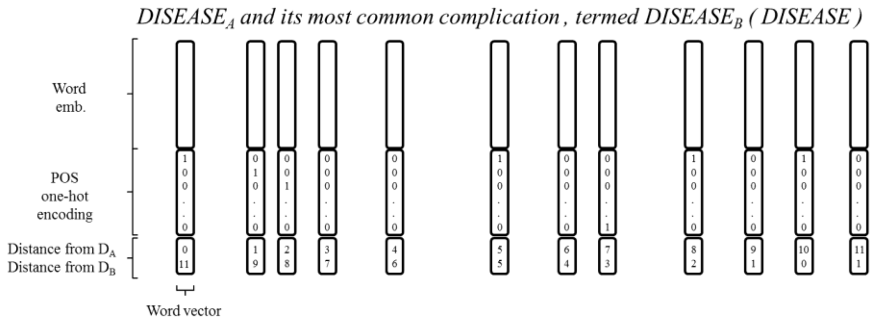

Supplement: Multimedia Appendix 2 [file medinform_v7i4e14502_app2.pdf]
