# Supplementary material for: Using a Large Margin Context-Aware Convolutional Neural Network to Automatically Extract Disease-Disease Association from Literature: Comparative Analytic Study
Source: JMIR Med Inform. 2019 Nov 26;7(4):e14502. doi: 10.2196/14502 (PMC6913619; doi:10.2196/14502)
Supplement: Multimedia Appendix 3 [file medinform_v7i4e14502_app3.pdf]

Multimedia Appendix 3 Context Representation Layer

Contextual information, such as pair and document information, is very useful for classification and has been widely used in previous research. The purpose of using contextual representation is to introduce traditional contextual features into a neural network architecture through simple representation. We can then apply the fully connected layer to the context vector to obtain a condensed vector that combines two different representations. Here are the features used in our contextual representation:

Bag-of-Words (BoW):

Word embedding has been shown to represent abstract information about words. However, word embedding can sometimes change the original meaning of a word. For example, "not" usually appears in negative DDA statements. However, in the word2vec model trained on GoogleNews, the three words most similar to "not" are "do", "did" and "anymore". This violates our intuition that “don't”, “doesn't” and “isn't” are more similar to “not” in the DDA statement. Since the embedded vector words of certain words may differ in the news and biomedicine domains, we use BOW features for context vector. Our BOW features include unigram, bigram, and surrounding diseases. All feature functions are shown in Table 1.

Part-of-speech (POS): The POS tags are commonly used for relation extraction. We use one-hot encoding to represent each word’s POS tag type.

NE Information: The number of diseases is useful when classifying DDAs. We use three different features to capture information, including:

• The number of tokens between disease pairs.

• The number of diseases between disease pairs.

• The number of diseases in the sentence.

Document-level information: Biological papers usually follow a certain flow to describe their experimental and scientific findings. Therefore, article structure often provides valuable information about DDAs. We use two types of article-level feature, core pair and pair location.

The core pair features indicate whether the current disease is a top-3 frequent disease pair in the article. The three most frequent pairs are treated as three features.

The pair location feature is used to indicate the position of the sentence containing the DDA in the article. If the sentence is the article title, it usually contains the subject of the article, which might be a DDA investigated in the paper. Similarly, if the sentence is the last sentence of the abstract, it may summarize the main scientific discovery of the article. We use three binary features to represent DDA pairs that appear in the title, the first sentence of the abstract, the last sentence of the abstract.

Table 1. Context Features.

**Name**	**Explanation**
Bag-of-word	
Inter word	Include the unigram, bigram and trigram words between disease pair
Surrounding word	Include the two words before the first disease NE and the two after the second disease
Inter word stem	Include the NLTK's stem of the inter word features
Surrounding word stem	Include the NLTK's stem of the surrounding word features
Part-of-speech	
Inter POS	Include the POS tags of the inter word features
Surrounding POS	Include the POS tags of the surrounding word features
NE information	
Distance between disease pair	It is the number of tokens between the disease pair.
Sentence disease count	It is the number of disease NEs in the sentence.
Between disease count	It is the number of disease NEs between the disease pair.
Document-level information	
Is it the most common pair	Whether the pair is the most frequent pair in the abstracts?
Is it the second common pair	Whether the pair is the second frequent pair in the abstracts?
Is it the third common pair	Whether the pair is the third frequent pair in the abstracts?
In title	Does the disease pair also appear in the title
In first sentence	Does the disease pair also appear in the first sentence
In last sentence	Does the disease pair also appear in the last sentence
