# Supplementary material for: Using a Large Margin Context-Aware Convolutional Neural Network to Automatically Extract Disease-Disease Association from Literature: Comparative Analytic Study
Source: JMIR Med Inform. 2019 Nov 26;7(4):e14502. doi: 10.2196/14502 (PMC6913619; doi:10.2196/14502)
Supplement: Multimedia Appendix 4 [file medinform_v7i4e14502_app4.pdf]

Multimedia Appendix 4: Hyperparameters

Table 1. Hyperparameters

	LSTM	BiLSTM	CNN	$CR_{cross\text{-}entropy}$	LC-CNN	SCNN	BERT	BioBERT
Word embedding size	300	300	300	300	300	300	768	768
Sequence length	160	160	160	160	160	160	128	128
Convolutional window size	-	-	5	-	5	5	-	-
Convolutional filter size	-	-	32	-	32	32	-	-
Batch size	8	8	8	8	8	8	32	32
Epoch	15	20	5	10	10	10	10	3
Dropout rate	-	-	0.5	0.2	0.2	0.2	0.5	0.1
