# Supplementary material for: Using a Large Margin Context-Aware Convolutional Neural Network to Automatically Extract Disease-Disease Association from Literature: Comparative Analytic Study
Source: JMIR Med Inform. 2019 Nov 26;7(4):e14502. doi: 10.2196/14502 (PMC6913619; doi:10.2196/14502)
Supplement: Multimedia Appendix 5 [file medinform_v7i4e14502_app5.pdf]

Multimedia Appendix 5: Performances on Tuning Set

Table 1. Performance of different models.

Method	P(%)	R(%)	F (%)
1. LSTM	65.53	70.15	67.76
2. BiLSTM	73.78	70.12	71.90
3. CNN	75.31	75.39	75.35
4. $CR_{cross\text{-}entropy}$	79.26	72.55	75.76
5. SVM	74.86	81.03	77.86
6. LC-CNN	80.42	88.28	84.16

Table 2. Performance of combined classifiers.

Model	Tuning set		
**Single stage**	**P(%)**	**R(%)**	**F(%)**
Baseline 1 ($CR_{cross\text{-}entropy}$)	79.26	72.55	75.76
Baseline 2 ($CR_{Hinge\text{-}loss}$)	74.86	81.03	77.86
Baseline 3 (CNN)	75.31	75.39	75.35
LC-CNN	80.42	88.28	84.16
**Two-stage**	**P(%)**	**R(%)**	**F(%)**
SVM + CNN	84.04	68.44	75.44

Table 3. The effect of different composite embedding vectors on tuning set performance.

Method	P(%)	R(%)	F(%)
$LC\text{-}CNN_{PubMed}$	81.19	87.36	84.07
$LC\text{-}CNN_{News}$	77.30	89.94	83.14
$LC\text{-}CNN_{no\ pre\text{-}train}$	75.17	88.95	81.48
$LC\text{-}CNN_{PubMed}$ - POS embedding	80.49	88.10	83.92
$LC\text{-}CNN_{PubMed}$ - NE distance embedding	82.13	85.34	83.56
