# Supplementary material for: Using a Large Margin Context-Aware Convolutional Neural Network to Automatically Extract Disease-Disease Association from Literature: Comparative Analytic Study
Source: JMIR Med Inform. 2019 Nov 26;7(4):e14502. doi: 10.2196/14502 (PMC6913619; doi:10.2196/14502)
Supplement: Multimedia Appendix 6 [file medinform_v7i4e14502_app6.pdf]

Multimedia Appendix 6: Generating Word Embedding

The following rules are used in our experiments to generate the word embedding model:

- We convert all words to their stems in lowercase.
- Although the performance of pre-trained embedding in LC-CNN is better than embedding trained in DDAE data, if pre-trained embedding is used, the vector corresponding to disease mention will vary with the word, such as *"hypertension"* and *"High blood pressure"* will correspond to different embedding vectors. So the LC-CNN classification model treats the two as different inputs, but humans treat these two situations as similar inputs. For the target pair of disease mentions, we set their embedding vectors as those of *"hypertension"* and *"diabetes"*.
- For other disease mentions, we set their vectors as the embedding vector of *"it"*.
- Words that cannot be found in the word embedding dictionary are removed.
