# Supplementary material for: Using a Large Margin Context-Aware Convolutional Neural Network to Automatically Extract Disease-Disease Association from Literature: Comparative Analytic Study
Source: JMIR Med Inform. 2019 Nov 26;7(4):e14502. doi: 10.2196/14502 (PMC6913619; doi:10.2196/14502)
Supplement: Multimedia Appendix 7 [file medinform_v7i4e14502_app7.pdf]

Multimedia Appendix 7: Architecture of $CR_{cross\text{-}entropy}$

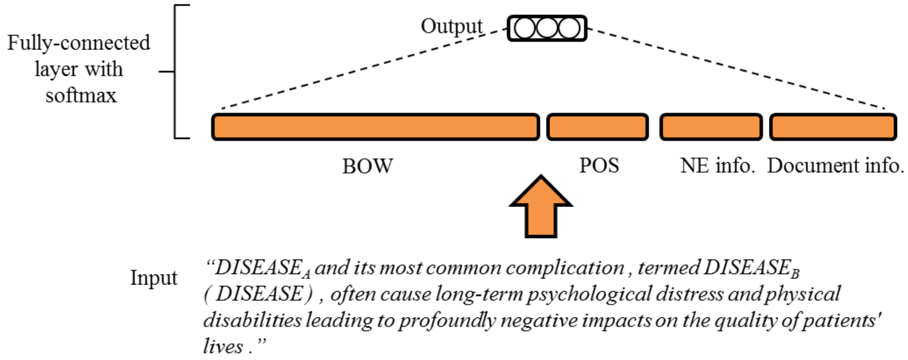

H. Architecture of SVM + CNN

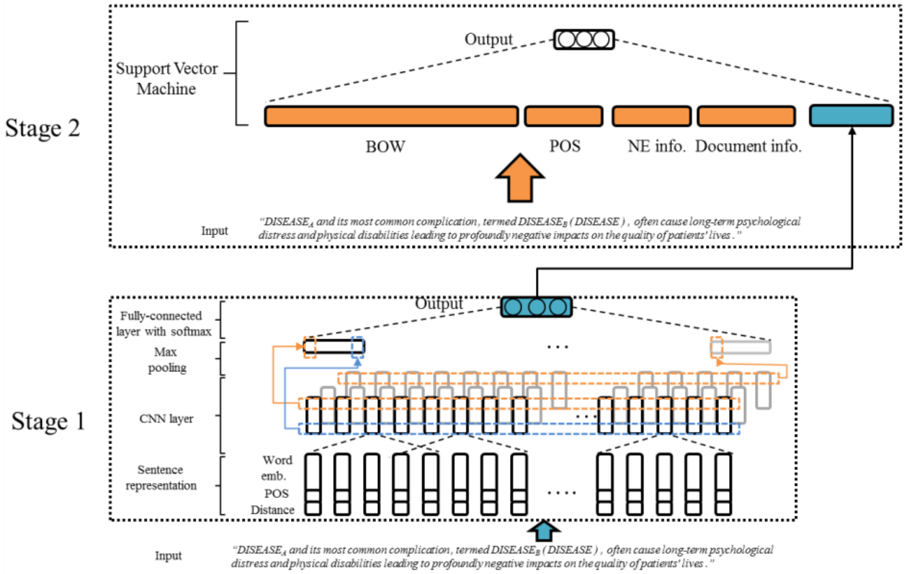

I. Effect of Different Features on LC-CNN

Table 3. The effect of different features on performance.

**Model**	**Tuning**			**Test**		
	**P(%)**	**R(%)**	**F(%)**	**P(%)**	**R(%)**	**F(%)**
LC-CNN$_{PubMed}$	81.19	87.36	84.07	**82.36**	85.00	**84.18**
- BOW	78.75	86.02	81.83	73.49	91.03	81.33
- POS	86.14	80.41	83.17	77.86	84.51	81.05
- NE information	76.20	91.27	83.06	74.49	87.85	80.62
- Document-level information	78.22	90.04	83.70	76.30	84.03	79.98
