# Supplementary material for: Using a Large Margin Context-Aware Convolutional Neural Network to Automatically Extract Disease-Disease Association from Literature: Comparative Analytic Study
Source: JMIR Med Inform. 2019 Nov 26;7(4):e14502. doi: 10.2196/14502 (PMC6913619; doi:10.2196/14502)
Supplement: Multimedia Appendix 8 [file medinform_v7i4e14502_app8.pdf]

Multimedia Appendix 8: Architecture of SCNN

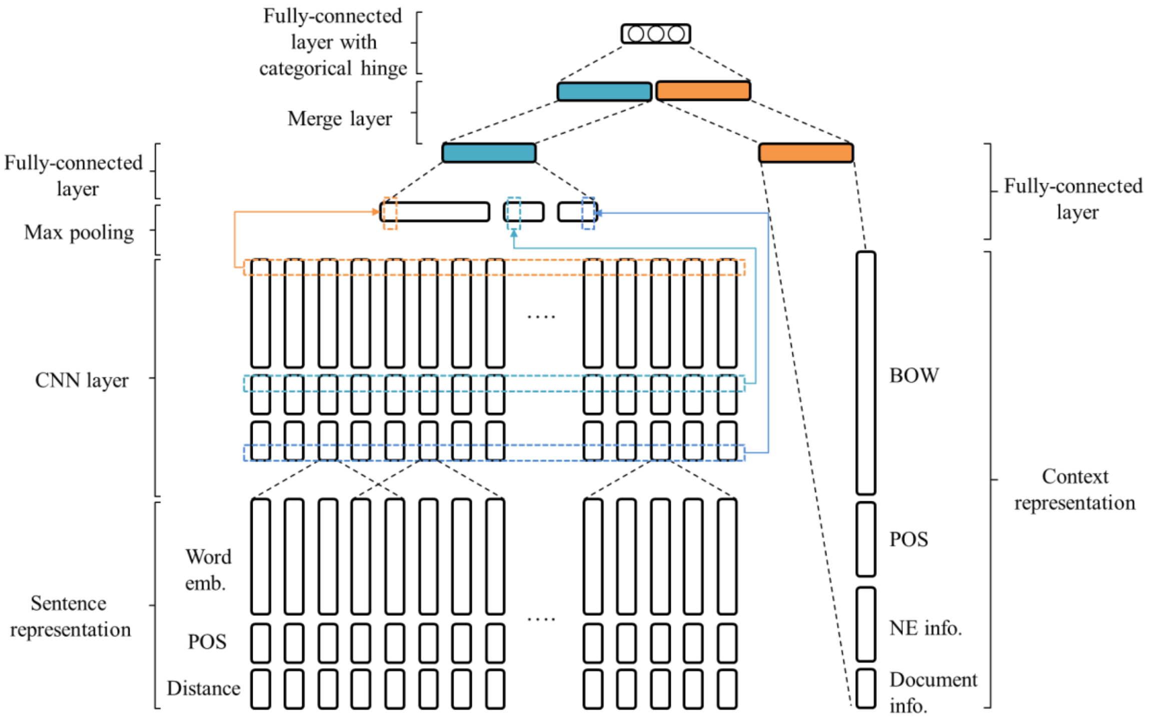

"DISEASE$_A$ and its most common complication , termed DISEASE$_B$ (DISEASE) , often cause long-term psychological distress and physical disabilities leading to profoundly negative impacts on the quality of patients' lives ."
