# Supplementary figures and images for: Using a Large Margin Context-Aware Convolutional Neural Network to Automatically Extract Disease-Disease Association from Literature: Comparative Analytic Study
Source: JMIR Med Inform. 2019 Nov 26;7(4):e14502. doi: 10.2196/14502 (PMC6913619; doi:10.2196/14502)

## Multimedia Appendix 9: Architecture of SVM + CNN

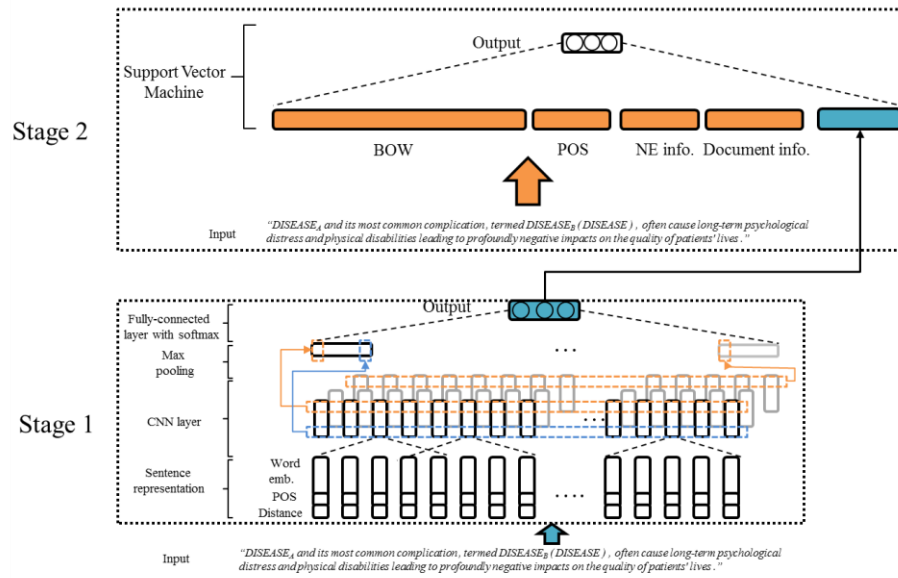

Supplement: Multimedia Appendix 9 [file medinform_v7i4e14502_app9.pdf]
